# Testing for normality in regression models: mistakes abound (but may not matter)

**DOI:** 10.1098/rsos.241904

**Published:** 2025-04-30

**Authors:** Stephen Midway, J. Wilson White

**Affiliations:** ^1^ Department of Oceanography & Coastal Sciences, Louisiana State University, Baton Rouge, LA, USA; ^2^ Department of Fisheries, Wildlife, and Conservation Sciences, Oregon State University, Newport, OR, USA

**Keywords:** regression assumptions, Shapiro–Wilk test, statistical power

## Abstract

This study examines the misuse of normality tests in linear regression within ecology and biology, focusing on common misconceptions. A bibliometric review found that over 70% of ecology papers and 90% of biology papers incorrectly applied normality tests to raw data instead of model residuals. To assess the impact of this error, we simulated datasets with normal, interval, and skewed distributions across various sample and effect sizes. We compared statistical power between two approaches: testing the whole dataset for normality (incorrect) versus testing model residuals (correct) to determine whether to use a parametric (*t*-test) or nonparametric (Mann-Whitney U test) method. Our results showed minimal differences in statistical power between the approaches, even when normality was incorrectly tested on raw data. However, when residuals violated the normality assumption, using the Mann-Whitney U test increased statistical power by 3–4%. Overall, the study suggests that, while correctly testing residuals for normality enhances model performance, the impact of testing raw data is negligible in terms of power loss, especially with large sample sizes. The findings highlight the need for more awareness of proper statistical practices, especially in evaluating the assumptions of linear models.

## 1. Introduction

The majority of commonly used frequentist, parametric statistical techniques depend on the assumption that data follow a particular probability distribution in order to calculate *p*-values. The normal distribution is the most commonly used probability distribution in this context, both because the central limit theorem applies to many scientific sampling processes and because maximum likelihood estimation can be done using the computationally simple least squares method. The importance and ubiquity of the normal distribution have led to the development of many statistical tests to quantify whether a sample of data has characteristics consistent with having been sampled from a normal distribution. Perhaps the first such test was Pearson’s Chi-Square [1], as it can compare observed and expected frequencies under a particular distribution (including the normal distribution). More formal normality tests were developed in the 20th century with the introduction of the Kolmogorov–Smirnov test [2] and the Shapiro–Wilk test [3], both of which are well-known and commonly used at the time of this writing. Since the mid-20th century, normality tests have proliferated; recent estimates suggested that at least 40 normality tests exist [4]. In that time, a number of studies have evaluated different sensitivities, power, and performance of normality tests (e.g. [5–7]), including several that used a normality test comparison to introduce their own normality test (e.g. [4,8,9]). Despite this array of testing procedures, a persistent question facing statistical practitioners, particularly in ecology, is when and why normality tests should be applied and the degree to which statistical inferences depend on the results of such tests.

One of the most common applications of normality tests is in the evaluation of assumptions in linear statistical models, including linear regressions and analysis of variance (ANOVA). The assumptions of linear regression are well known: that the relationship between the response and predictor is linear, and the residual errors are independently and identically distributed with a mean of 0, a constant variance (homoscedasticity), and follow a normal distribution [10]. Potential violations of this final assumption of normality garner considerable attention, and authors in a range of journals often report evaluating whether that assumption is met. Indeed, studies going back to the mid-20th century (e.g. [11–13]) have addressed the violation of the normality assumption (among other regression assumptions and conditions), yet more recent studies continue to point out that normality is often an overlooked assumption in regression analysis [14,15]. With linear regression techniques continuing to grow in popularity, use and development (e.g. generalized linear models, hierarchical models), uncertainty about how violations of model assumptions affect statistical inferences remains a concern.

An odd twist to conversations about addressing model assumptions is that there is a widespread misconception about what quantities need to be normally distributed in a valid regression model. Although textbooks and many other sources correctly explain that the model error terms (i.e. residuals) should be normally distributed, there is a commonly held—and incorrect—belief that the underlying raw data being modelled must follow a normal distribution. We base this statement on our own experience as reviewers and editors for ecological journals, but Kéry and Hatfield [16] also noted that testing (raw) data for normality is ‘by far the most common misconception we have come across…in general linear models’. Although we are not sure where this misconception came from, it is common for studies to report that they tested their data for normality (e.g. [14]). Even more concerning is that this incorrect assumption continues to be promoted in the published literature (e.g. [17,18]); Marmolejo-Ramos and González-Burgos [19] go so far as to say that parametric tests require ‘observations follow a bell-shaped distribution and that they peak around the mean’.

Ultimately, concerns about applying normality tests and whether model residuals are normally distributed are related to statistical inferences about the linear model. If the residuals are not normally distributed, is the associated *p*-value incorrect? Is it better to use a non-parametric test in such cases? Do parametric tests or nonparametric tests provide more statistical power to reject a false null hypothesis in those circumstances? Additionally, if a normality test is used inappropriately (e.g. by testing raw data instead of model residuals), how does that affect the analysis? We address those questions here in an effort to characterize the degree to which scientists should be concerned about the normality assumption in linear models. We address this issue in the context of null hypothesis testing using *p*-value cutoffs; that is, rejecting a null hypothesis if *p* < 0.05. There are many valid reasons to avoid the cutoff approach [20,21], but see Murtaugh [22] for why it provides a straightforward way to compare the ability of different approaches to detect true differences between sampled populations (i.e. the Type II or false negative error rate).

In this study, we had two objectives. First, we conducted a bibliometric review to estimate the specific types of normality tests being used in peer-reviewed articles in ecology and biology and to assess the appropriateness of their usage—e.g. whether they tested raw data or residuals for normality. The second objective was to conduct a simulation analysis that evaluated the power to detect a statistical difference between two samples when the data have either normally- or non-normally distributed residuals. In doing this we simulated common statistical workflows, including the application of both parametric linear models and a nonparametric alternative, and evaluated when deviations from normality reduced statistical power.

## 2. Material and methods

### 2.1. Bibliometric review

We conducted a bibliometric literature review to quantify the use of several popular normality tests. The objective of this review was to first describe what the current conventions are for normality tests in biology and ecology, and second, to inform some of the tests we would adopt for later simulation modeling. We conducted a literature search on 23 October 2023 to enumerate putative uses of nine different normality tests reported in the ecological literature (see table 1 for the lists of tests). The search was conducted in Google Scholar because Web of Science (and comparable bibliometric databases) often does not search the full text of articles, and normality tests are not commonly included in the title, abstract or other searchable article metadata. As important as they may be, normality tests are often diagnostic and not a primary methodology that warrants high-level reporting. Although Google Scholar searches the entire article text, there were still some search limitations. For example, we were not able to search by discipline or category of journals, because these fields do not exist as search options. As a workaround, we specified our search parameters so that only journals with the term *biology* or *ecology* in the title were searched. We recognize that this is an imperfect method to exhaustively search the full disciplines of biology and ecological journals. Nevertheless, this bias was imposed on all searches and the results likely included enough journals that we expect to have detected the general trends within the disciplines. We also recognize that normality tests are widely used in many fields beyond biology and ecology; however, we wanted a field that was large enough to (likely) have all tests represented, while still confining our search to a specific discipline. (In addition, the study authors operate in the fields of biology and ecology, so we felt most comfortable working with this literature.) For each search, we used the name of the test in addition to the term *normality*, because some of the tests have applications beyond normality testing.

**Table 1.** Percentage of usage results from a bibliometric search (in October 2023) in Google Scholar for frequencies of normality tests appearing in published literature. The total number of reported uses of all terms by discipline (biology or ecology) are reported at the bottom of the table. The bold text in the 'test' column indicates the word used as search term, along with *normality*.

test	biology	ecology
Shapiro-Wilk test	50%	55%
Kolmogorov-Smirnov (K-S) test	32%	34%
Lilliefors corrected K-S test	3%	5%
Anderson–Darling test	3%	3%
D’Agostino skewness test	8%	2%
Jarque–Bera test	1%	1%
D’Agostino-Pearson omnibus test	3%	1%
Cramer-von Mises test	0%	0%
Anscombe–Glynn kurtosis test	0%	0%
Total reported uses (*n*)	17 877	8388

After doing a search for frequencies of normality tests, we selected the Shapiro–Wilk test [3] for further investigation. Specifically, we repeated the search described above for uses of the Shapiro–Wilk test of normality, but this time limited the search to only peer-reviewed studies published in 2022. The reason for this was (i) to limit the overall number of search results, (ii) have the search results represent usage in the current literature, and (iii) evaluate the use of the test (i.e. what is being tested for normality?) based on a range of journals. We reviewed the first 50 studies in the search results (as reported by Google Scholar based on relevance) in both biology and ecology that were in Web of Science Indexed journals and were available as full text to the authors. We evaluated the use of the test based on language in the study that described whether the Shapiro–Wilk test was used before any linear model, such as testing the response or predictor variables (i.e. the raw data) for normality, or whether the Shapiro–Wilk test was used after any linear model, such as to evaluate the model residuals for normality. Based on best practices for linear modeling, we classified the use of normality tests on the raw data as *inappropriate* and use of normality tests on the model results as *appropriate*.

### 2.2. Simulation study

#### 2.2.1. Baseline normality comparisons

We designed a simulation study to evaluate the effects of different approaches to using normality tests in a typical linear regression workflow. First, prior to testing any workflows, we simply sought to quantify the performance of common normality tests to understand baseline detection rates of different normality tests on a single sample from different data types. For this baseline comparison, we generated nine types of simulated data (with 10 000 data sets per simulation) representing all combinations of three sample sizes (*n* = 10, 20 and 50) and three types of true underlying data distributions (normal, interval and skewed). The normal distribution was specified with a mean of 0 and standard deviation of 1, the interval distribution was specified with a lower bound of −2 and upper bound of 2, a mean of 0 and a standard deviation of 1.5, and the skewed (lognormal) distribution was specified with a mean of 0 and standard deviation of 1, both of which are on the log scale. For each data set, we tested the null hypothesis that the sample was drawn from a normal distribution using the Shapiro–Wilk test [3], the Kolmogorov-Smirnov test [2], the Anderson–Darling test [23] and the Lilliefors test [24], four of the most commonly used normality tests in biology and ecology. Although we did not have strong hypotheses about these tests, we did expect to see some differences. For instance, the Kolmogorov-Smirnov test is based on expectations about the asymptotic behaviour of the maximum distance between an empirical distribution function and the normal cumulative distribution, and thus should only be reliable for large sample sizes; the Lilliefors test was designed as an improvement of that testing approach. By contrast the Shapiro–Wilk test compares the empirical distribution to the theoretical expectation across the entire distribution and is generally considered one of the most powerful normality tests. The Anderson–Darling test is conceptually similar but uses a weighting function to place more weight on the tails of the distribution, so could be expected to perform better on skewed distributions.

#### 2.2.2. Simulation design

**Figure 1.**
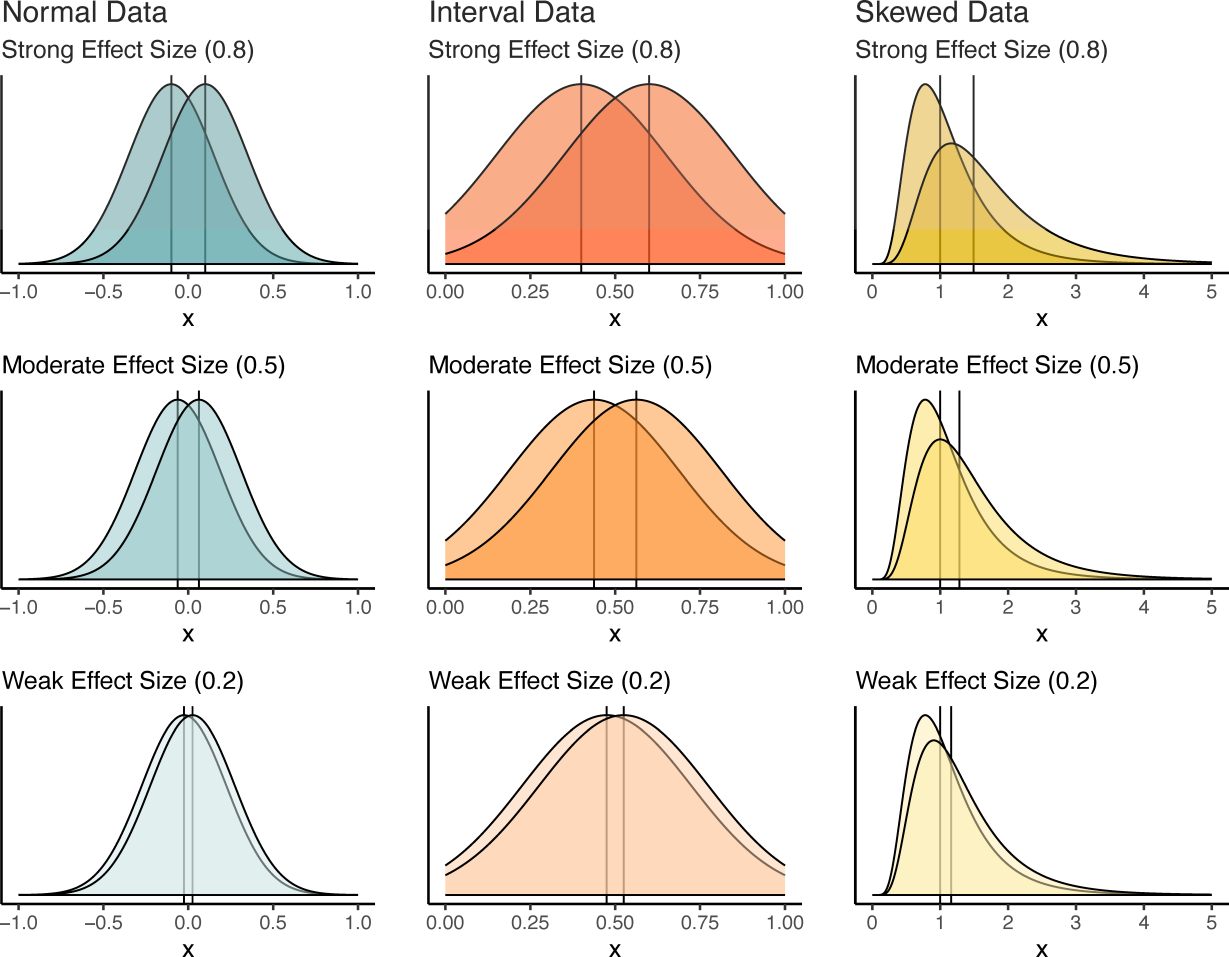
Visual representations of the distribution types (normal, interval and log-normal) used in *t*-tests and Mann-Whitney tests with data generated from three different effect sizes. The scenario of no effect (effect size = 0) is not shown, although it was included in the study.

After comparing the overall performance of the different tests, we wanted to create a simulation approach that better captured the use and workflow of normality testing in linear regression. To do this, we generated random samples of data from three sets of distributions (figure 1). The first distribution was a normal distribution that was included as a control group. The second distribution we examined was for interval data, which we generated from a truncated normal distribution bounded by 0 and 1. Interval data may appear to be normal when the mean is near 0.5 and the standard deviation is small, but the tails are truncated at 0 and 1 rather than extending from -∞ to ∞ as a normal distribution does. Interval data often arise in ecology (e.g. survival rates) and there is a long history of applying transformations to such data [25], in order to apply least squares methods that require the assumption of a normal error distribution. The third distribution we evaluated was for skewed data, which we generated from a log-normal distribution that had one long asymmetric tail. Log-normal distributions are also common in nature [26] and are often transformed to reduce skewness prior to using least squares methods.

**Figure 2.**
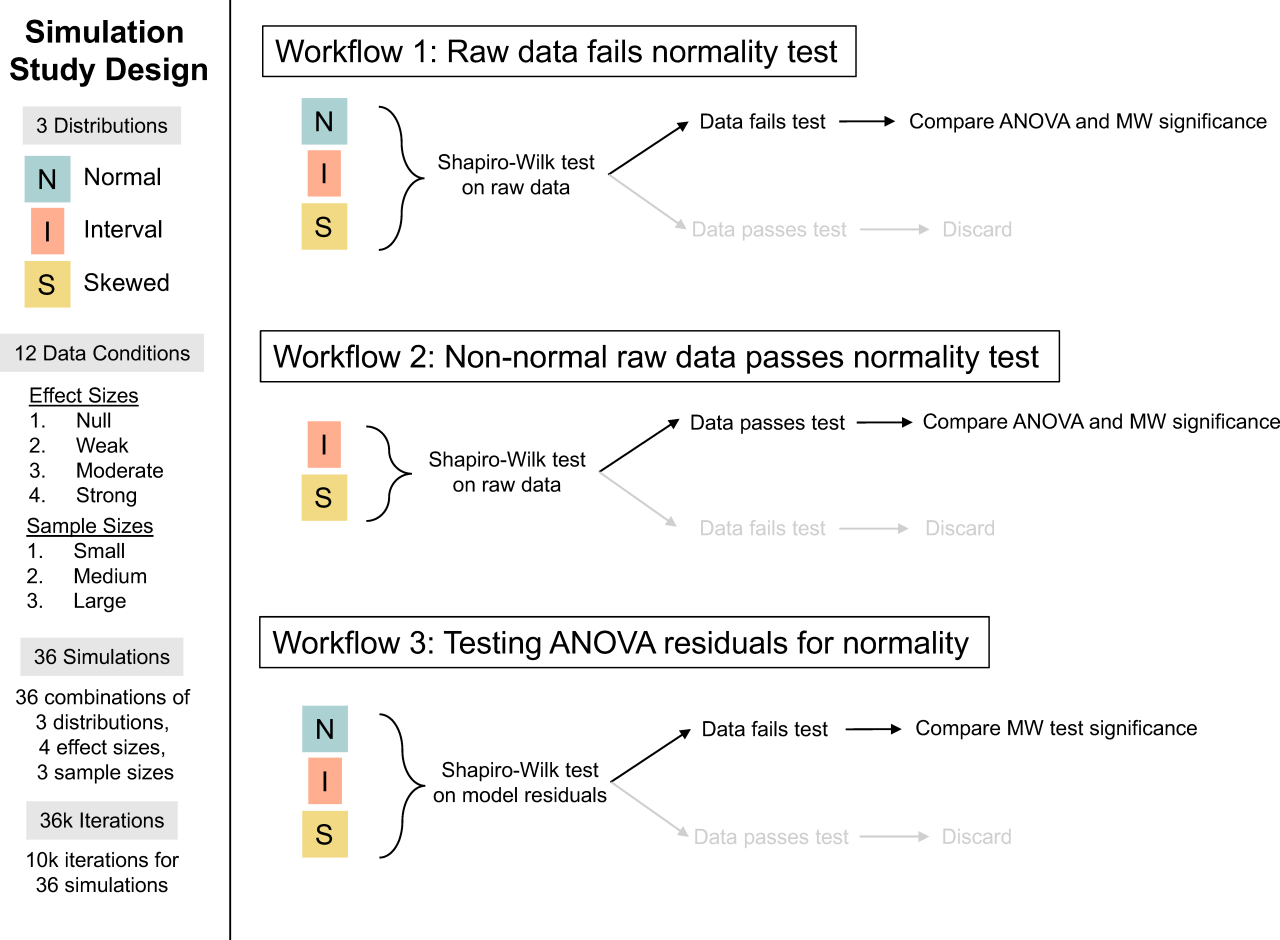
Summary of the simulation and Analytical Decisions used in the study. The simulation design is described along the left side, and the three Analytical Decisions that were used with the simulation data are described from top to bottom (MW = Mann-Whitney test).

For each of the three distributions, we created simulations that varied in sample size and effect size, both of which affected the power of statistical tests. Each simulated comparison used a dataset with samples drawn from two different groups and tested the null hypothesis that the two groups had different true means. We varied sample size such that groups had *n*_group_ = 10, 20 or 50 samples. *n*_group_ = 10 was included to represent a realistically low group sample size for study. *n*_group_ = 20 represented a medium-sized group; *n* = 20 is around the size where the *t* and *z* distributions become similar. Finally, *n* = 50 was used as the large sample size group, beyond which (e.g. *n*_group_ > 50) we would not expect simulation results to be particularly sensitive to sample size. For effect size, we used the well-established Cohen’s *d* = 0, 0.2, 0.5 and 0.8, which correspond to no effect (i.e. the null hypothesis is true), and weak, moderate and strong effect sizes [27]. Because we simulated data with the same standard deviation, we calculated Cohen’s *d* with a pooled standard deviation (among groups) and focused on manipulating the mean differences between the two groups in order to change the effect size (figure 1). This led to 36 different simulation scenarios: four effect sizes and three sample sizes for each of three distributions (see figure 2 for the simulation study design).

We performed 10 000 replicate simulations for each of the 36 scenarios (resulting in *n* = 36 000 iterations for the entire study). Each iteration followed the same set of analytical decisions, which we designed to mimic the way ecologists often apply tests for normality (figure 2). We summarize the steps here and then give further details in the next subsection. First (Analytical Decision 1), the two samples in the simulated dataset were pooled and tested for normality using the Shapiro–Wilk test, and we recorded whether the dataset 'passed' (i.e. did not reject the null hypothesis that the pooled dataset was drawn from a normal distribution) or 'failed' (i.e. the null hypothesis of normality was rejected) the test. The Shapiro–Wilk test was selected over other normality tests because of its power and common usage; we wanted our simulations to reflect common protocols. Next (Analytical Decision 2), we performed both a *t*‐test and a nonparametric Mann-Whitney U test [28] to test the null hypothesis that the means of the two groups did not differ. We used a *t*‐test as it is the two-sample version of a linear model and shares the same assumptions as ordinary linear regression and analysis of variance. The Mann-Whitney U test is a nonparametric rank-based test; it is often used as a nonparametric alternative to the *t*‐test although technically it is testing the null hypothesis that the two samples are drawn from the same distribution, not that they are drawn from populations with the same mean. In any case, it can be used to make similar inferences, and when residuals are normally distributed it has an 'efficiency' that is 95% of the *t*‐test (i.e. probability of correctly rejecting a false null hypothesis; [29]). For each of the simulated datasets, we recorded the *p*-values and the associated determinations of statistical significance (i.e., reject the null hypothesis or not), based on an α = 0.05, from each of the two tests. Finally (Analytical Decision 3), we tested the residuals from the *t*‐test for normality using the Shapiro–Wilk test and recorded whether the test rejected (α = 0.05) the null hypothesis of normally distributed residuals. This same set of procedures and tests was applied to each dataset. This led to different possible analytical pathways based on the results of those tests, corresponding to three common sequences of tests that investigators follow when using normality tests; we refer to these pathways as Analytical Decisions.

#### 2.2.3. Analytical Decision 1: raw data fails normality test

Analytical Decision 1 (figure 2) followed an analysis sequence in which the raw data were pooled across groups and tested for normality, and the null hypothesis that the data were normally distributed was rejected. When this approach is followed, the investigator would typically then use a non-parametric test to evaluate the null hypothesis that the two samples are from different populations. Testing for normality in this way is not the correct or recommended procedure, but it is a very common approach, so we wanted to understand what consequences this approach would have for the overall test for differences in sample means. Therefore after 'failing' the Shapiro–Wilk test for normality, each data set underwent both a *t*‐test and Mann-Whitney U test and we then compared the error rates by effect size, sample size, distribution, and test type. Scenarios with effect size of 0 (no effect) were used to calculate Type I errors (probability of rejecting a true null hypothesis, or false positive), while all other scenarios were evaluated in terms of the Type II error rate (probability of failing to reject a false null hypothesis, or false negative).

#### 2.2.4. Analytical Decision 2: non-normal raw data passes normality test

Analytical Decision 2 (figure 2) followed an analysis sequence that is the alternative pathway to Analytical Decision 1: the raw data was tested for normality and 'passed', leading to a parametric test (*t*‐test) for differences in sample means. In this analytical decision, we only examined the datasets from the interval and skewed datasets, so we dealt with the case when the data were actually non-normally distributed but nonetheless 'pass' a normality test. We excluded the normal distribution from this analytical decision because the intention was to examine data from non-normal distributions that passed a normality test. As in Analytical Decision 1, testing the raw data for normality is not the recommended procedure, but we include it because it is a very common approach that we want to better understand in terms of errors and outcomes. For the simulations that passed the Shapiro–Wilk test for normality, each data set underwent both a *t*‐test and Mann-Whitney U test and we then compared error rates as described in Analytical Decision 1.

#### 2.2.5. Analytical Decision 3: testing ANOVA residuals for normality

Analytical Decision 3 (figure 2) was designed to represent an appropriate use of normality tests, in which a *t*‐test is done on all simulated data sets, followed by a normality test on the model residuals. For datasets in which the model residuals failed a Shapiro–Wilk test (i.e. were determined to be non-normal), we compared the Type II error rate of the *t*‐test to that of the Mann-Whitney U test. When model residuals fail a normality test, there may be several options, but using a non-parametric (e.g. Mann-Whitney U) is a common option, and we wanted to evaluate whether the Mann-Whitney U test resulted in greater statistical power when the assumptions of normally distributed *t*‐test residuals were not met.

Note two important considerations about the simulations. First, although the Kolmogorov-Smirnov test was a very popular normality test based on the literature, we only included it in the baseline normality comparison and not the simulation study. We excluded it because it is not recommended for sample sizes < 50 [8,30]. It is extremely conservative and in our exploratory tests, it found nearly all datasets we tested to be non-normal, unlike the other three normality tests we used, which performed similarly to each other.

## 3. Results

### 3.1. Bibliometric review

Frequencies of specific normality tests were comparable in both biology and ecology, with most tests being about twice as frequent in biology as in ecology, which is likely attributable to the discipline of biology being larger than ecology (table 1). (Note the D’Agostino skewness test and D’Agostino-Pearson omnibus test were extremely more frequent in biology than ecology, but this may be explained by their uses beyond normality testing—despite our use of the term *normality* in the search.) In both disciplines, the Shapiro–Wilk test was the most commonly used test, followed by the Kolmogorov-Smirnov test. All other tests were reported at relatively low frequencies.

Based on 50 papers published in 2022 in both biology and ecology journals, we found the vast majority of studies using normality tests with linear models were using them inappropriately (paper details are in electronic supplement S1). Of the papers we reviewed, 90% of those in biology journals and 70% of those in ecology journals reported using the Shapiro–Wilk normality test on the data *before* doing any linear regression, and the outcome of the test often directly informed the subsequent statistical test (i.e. parametric vs non-parametric test). Impact Factors of journals represented ranged from 1.6–11.6 in biology and 1.4−8.8 in ecology, with no apparent trend of usage in relation to Impact Factor (likely because there were so few correct uses).

Given the prevalence of incorrect normality testing in the biology and ecology literature, it is important to evaluate whether these misapplications meaningfully impact the validity of statistical inferences. If incorrect normality testing leads to (substantial) changes in power or error rates, it could indicate a widespread problem in ecological and biological research. Conversely, if the consequences are minimal, it would suggest that while incorrect, these practices may not necessarily undermine or alter conclusions.

### 3.2. Simulation study

#### 3.2.1. Baseline normality comparisons

**Figure 3.**
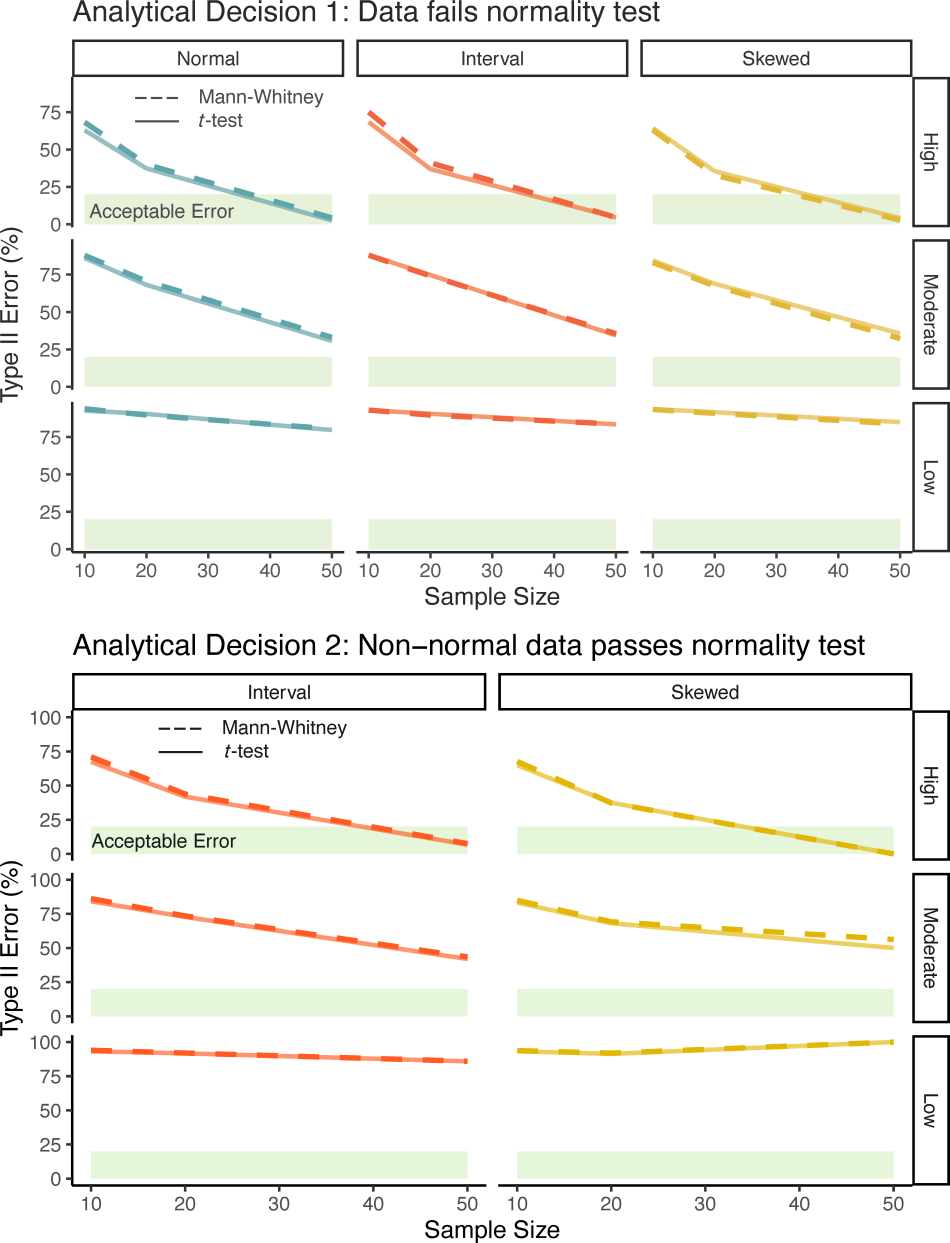
Type II error rates (%) for Mann-Whitney U test and *t*‐test for Analytical Decisions 1 and 2. Panels within each Analytical Decision show combinations of the different baseline distributions that were used to generate the data, the effect size (right axis), and the sample size (bottom axis). Acceptable error is represented by the light green area.

**Figure 4.**
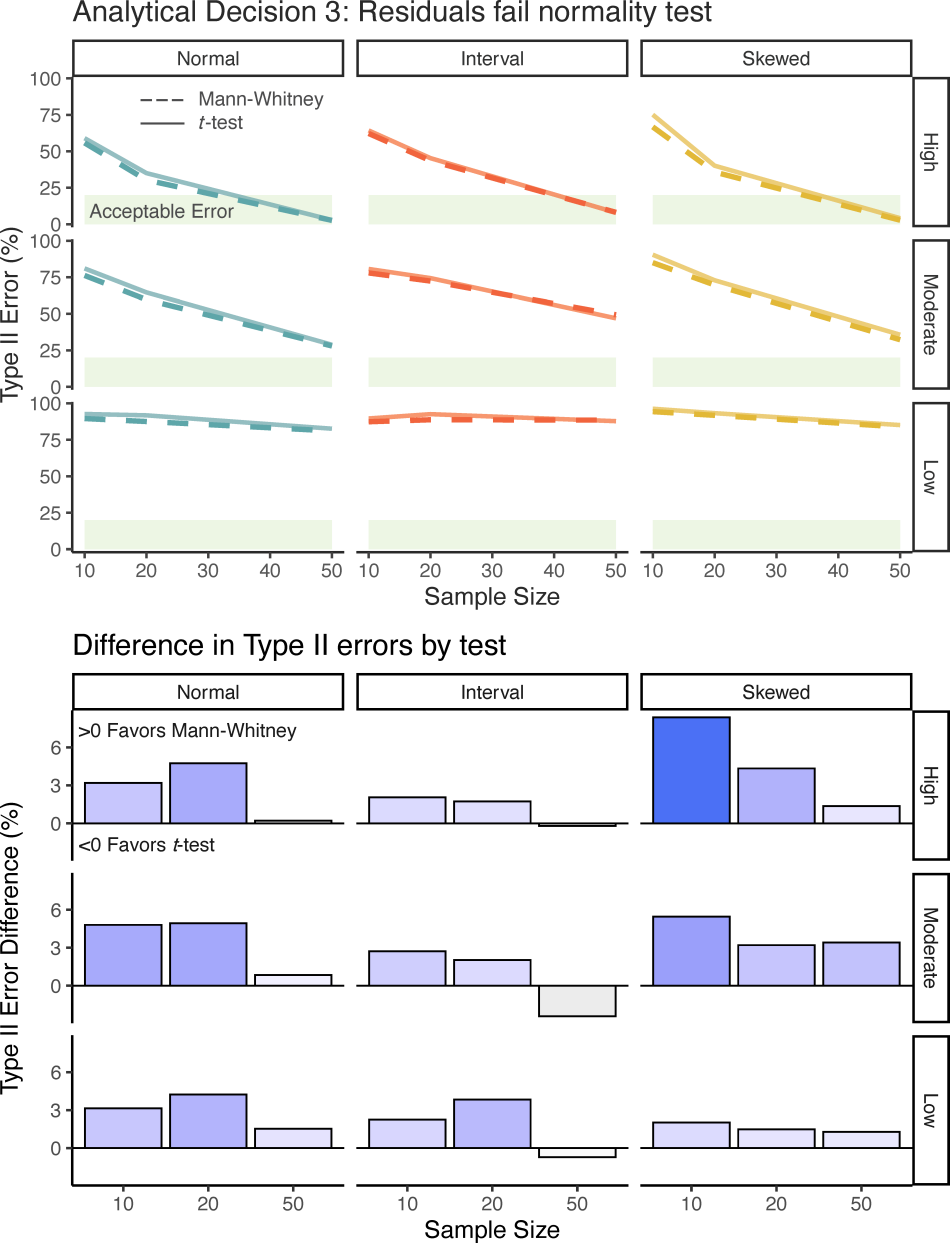
Top panel: Type II error rates (%) for Mann-Whitney U test and *t*‐test for Analytical Decision 3. Panels within each Analytical Decision show combinations of the different baseline distributions that were used to generate the data, the effect size (right axis), and the sample size (bottom axis). Acceptable error is represented by the light green area. Bottom Panel: Differences in Type II error rates when comparing Mann-Whitney U test and *t*‐test for Analytical Decision 3. Positive differences are shown in blue bars and represent better performance by Mann-Whitney U test, while negative differences are shown in gray bars and represent better performance by a *t*‐test.

Our first analysis evaluated how much agreement there was among normality tests and how tests were performed under sample sizes and distributions. When applied to samples drawn from normal distributions, all tests produced *p*-values that failed to reject the (true) null hypothesis of normality 95% of the time, which is consistent with the intended Type I error rate of 5% (table 2). When data were drawn from an interval distribution, the tests failed to reject the false null hypothesis of normality in 75−96% of simulations. Although there were slight differences in that proportion among the different normality tests, there was a clearer pattern with respect to sample size, with all tests producing consistently smaller *p*-values as sample size increased. In general, datasets with smaller sample sizes drawn from interval distributions are less likely to include extreme values near the 0 and 1 limits and may, therefore, more closely resemble data sampled from a normal distribution, while datasets with larger sample sizes are more likely to have well-defined bounds of the interval, providing greater evidence of deviation from normality (figures 3 and 4). Simulations with data drawn from skewed distributions 'failed' all of the normality tests at a higher rate than data drawn from interval distributions, though like interval data there was a strong effect of sample size increasing the power to detect deviations from normality (figures 3 and 4). For the smallest sample size (*n* = 10), data drawn from skewed distributions 'passed' normality tests approximately half the time (39−53%, for all tests except Kolmogorov-Smirnov, which had a 0% rate of failing to reject the null hypothesis of normality), while for the largest sample size (*n* = 50), the null hypothesis of normality was rejected for all of the simulated datasets, using all of the normality tests. For the datasets drawn from interval and skewed distributions, the Lilliefors test showed a slight, but consistently higher rate of failing to reject the null hypothesis of normality, relative to the Shapiro–Wilk and Anderson–Darling tests. Overall, the Shapiro–Wilk test had the lowest false negative error rate across the different distributions and sample sizes, so used it as the primary normality test for subsequent analysis. This was also consistent with additional recommendations from other studies [7,8,30].

**Table 2.** Baseline normality test performance. Cell values are the percent of 10 000 simulated datasets for each combination of distribution and sample size that passed each of four different normality tests. (Here, 'passing' a normality test means that the test failed to reject the null hypothesis that the sample data were drawn from a normal distribution).

distribution	sample size (*n*)	Shapiro–Wilk	Kolmogorov-Smirnov	Anderson–Darling	Lilliefors
normal	10	95%	95%	95%	95%
normal	20	95%	95%	95%	95%
normal	50	95%	95%	95%	95%
interval	10	96%	93%	95%	96%
interval	20	93%	92%	93%	95%
interval	50	75%	90%	82%	91%
skewed	10	39%	0%	42%	53%
skewed	20	7%	0%	10%	21%
skewed	50	0%	0%	0%	0%

#### 3.2.2. Analytical Decision 1

For the simulated datasets drawn from normal and interval distributions, only about 5% were found to be non-normal (Shapiro–Wilk *p*‐value < 0.05), with a slightly higher rate of detecting non-normality (15%) for datasets with large sample size and large effect sizes. (Even data from normal distributions was more often identified as non-normal in cases of large effect sizes, because the combined samples likely created a bimodality that made it harder to detect normality.) Skewed data simulations failed normality tests at a high rate, ranging from 20 to 99% failure across nine simulations. For simulated datasets from all three distributions, the Type I (false positive) error rates calculated on simulations with effect sizes of 0 were similar and ranged from 4−6%, very close to the intended rate of 5%. For the 27 simulation scenarios that had effect sizes > 0, the patterns of Type II error (false negative; failing to detect non-normality) were similar across the three types of distributions and decreased with increasing sample sizes and effect sizes.

The premise of this Analytical Decision was that an investigator has incorrectly applied a normality test to raw data, and then chose to use a nonparametric test for the null hypothesis that the two samples are from different populations. This would be a concern if the nonparametric test had less power to detect true differences in this context. However, in scenarios with effect sizes >0 (i.e. the null hypothesis is false), the *t*‐test and Mann-Whitney U test had very similar Type II error rates (figure 3*a*). For datasets drawn from normal or interval distribution, the *t*‐test Type II error rates were slightly lower by an average of 1.9% and 1.3%, respectively. Skewed data showed lower Type II error rates for the Mann-Whitney test, although only by an average of 1.5%. Overall, the Type II error rates in this Analytical Decision were similar between the *t*‐test and Mann-Whitney U test, regardless of the overall error distribution. The tests only produced a Type II error rate < 0.2 (corresponding to power of 0.8) with a large effect size and sample size of *n* > 30.

#### 3.2.3. Analytical Decision 2

In Analytical Decision 2, we examined the simulated datasets drawn from non-normal distributions that 'passed' a normality test on the raw data (i.e. the set of simulations excluded from Analytical Decision 1, but without those actually drawn from a normal distribution). For the 24 scenarios investigated in this Analytical Decision, the number of simulated datasets that passed the normality test was extremely variable. A total of 79−97% of simulated datasets drawn from interval distributions passed normality, but only 11−48% of simulated datasets drawn from skewed distributions with the low and moderate sample sizes passed, while < 1% of skewed simulated datasets with large sample sizes passed the normality test (between 7−16 of 10 000 simulations).

In scenarios with effect size = 0 (null hypothesis was false) the Type I error rates (false positive) were consistently between 4.9% and 5.4% for both distribution types and most sample sizes. The exception was that for datasets with large sample size drawn from a skewed distribution, the Type I error rate was 0%, a function of the fact that only 8 simulations out of 10 000 were included in this Analytical Decision.

The pattern of Type II error rates (false negative) for the 18 scenarios with an effect size > 0 was nearly identical to that in Analytical Decision 1, meaning that for a given effect size and distribution, the Type II error rates tended to decrease substantially with sample size, but with weaker decreases for lower effect sizes. This pattern was very similar for the two different distribution types. Overall, the average Type II error rates were lower when using the *t*‐test than the Mann-Whitney U test; however, as in Analytical Decision 1, the differences were small (1.3 and 1.4% for the interval and skewed distributions, respectively).

#### 3.2.4. Analytical Decision 3

Approximately 5−10% of simulated datasets drawn from normal and interval distributions had residuals that failed the Shapiro–Wilk test, whereas datasets drawn from the skewed distribution had residuals that failed the Shapiro–Wilk test at a high rate, ranging between 45 and 99% across simulation combinations. Applying the Mann-Whitney U test to these datasets consistently provided more power to detect differences between the groups (lower Type II error; note that this is a difference from the results in Analytical Decisions 1 and 2). For datasets drawn from the interval distributions, the Mann-Whitney test only decreased the Type II error rate by 1.3%, but in scenarios with data sampled from the normal and skewed distributions, there was on average 3.1 and 3.4% (respectively) lower Type II error using the Mann-Whitney test. One exception to the improved performance of the Mann-Whitney test was the simulations with large sample size datasets drawn from interval distribution, which showed weak evidence for the *t*‐test having a lower Type II error rate.

## 4. Discussion

Our analysis of simulated datasets drawn from both normal and non-normal distributions indicates that there is little difference in statistical power between parametric linear models and their non-parametric equivalents, regardless of the underlying distribution of the data. We considered two basic approaches to concerns about data distributions. The first is commonly used, even though technically incorrect: test the underlying raw data for normality before deciding whether to use a parametric linear model or a non-parametric test. We found that regardless of the results of such a test, and regardless of the actual distribution of the data, the parametric *t*‐test had nearly identical power to the non-parametric Mann-Whitney test. The key factors affecting the power to detect differences among group means were the sample size and effect size, not the test or actual distribution. The second approach we considered is a recommended best practice, which is to examine residuals for normality after fitting a linear model. In that case we did find that if the residuals were found to deviate from normality, using a non-parametric test provided greater power to detect differences in sample means, but the effect was small (approx. 3%). Therefore, we find little cause for concern about mistaken use of normality tests prior to model fittings, as well as renewed confidence in the robustness of parametric linear models to deviations from the assumption of normally distributed residuals.

### 4.1. Analytical Decisions

While all normality tests performed similarly when data were normally distributed, discrepancies emerged for non-normal data. The Shapiro–Wilk test consistently performed better, especially for skewed distributions, where it outperformed alternatives like the Kolmogorov-Smirnov and Anderson–Darling tests. The Shapiro–Wilk test is sensitive to deviations from normality, particularly in smaller sample sizes and non-normal distributions that likely improve its performance over other tests. In contrast, tests like the Kolmogorov-Smirnov are more conservative, and often failed to reject the null hypothesis for non-normal data, especially in small samples. We expected the Shapiro–Wilk test to be one of the most reliable and versatile normality tests, and this suspicion was well supported and confirmed our use of Shapiro–Wilk in the simulations.

Analytical Decision 1 represents an inappropriate use of a normality test, because the normality test is used on the raw data and not the model residuals. In cases where the raw data fails in a normality test, many users would pivot to a Mann-Whitney U test, and this Analytical Decision was designed to understand the differences in statistical power when different tests are employed in this scenario. Our scenarios found strong evidence that the Mann-Whitney test did not provide any increase in statistical power. In fact, only for skewed data did we see a very marginal increase in power over a *t*‐test, while the *t*‐test was slightly more powerful for the scenarios derived from the normal and interval distributions. By far, increasing the group sample size, particularly with the high and moderate effect size, was the greatest way to reduce Type II errors. Although we do not recommend ever using a normality test on the raw data, there appears to be little consequence in terms of statistical power between a parametric or non-parametric analysis of the data when the raw data are inappropriately tested for normality. Findings from Analytical Decision 1 suggest that studies using the approach of testing the raw data for normality, while flawed in their methodology, may still produce valid results and conclusions. Our findings, however, do not justify the practice. We emphasize that more thought and focus should be placed on model design and validation instead of worrying about the distribution of the input data.

Analytical Decision 2 represented a second scenario where a normality test is inappropriately used on raw data. However, unlike Analytical Decision 1, Analytical Decision 2 captures the cases when data from non-normal distributions passes the normality test. In such cases, a *t*‐test would almost always be used because passing the normality test would not suggest a non-parametric alternative. Our results suggest that although Type II error rates were on average lower with the *t*‐test than with the Mann-Whitney test, the differences were marginal and similar to Analytical Decision 1, a *t*‐test or a Mann-Whitney test would likely produce similar outcomes with little consequence of choosing one test over the other. This result reveals another analytical consideration: researchers relying on normality tests of raw data may be falsely reassured that parametric methods are appropriate. While our results show little impact on power, they highlight how statistical misconceptions can shape analytical workflows. Addressing this misconception would lead to better statistical habits and a stronger emphasis on residual diagnostics rather than arbitrary normality tests.

Analytical Decision 3 was our only simulation of an appropriate application of a normality test in linear regression, and this was the Analytical Decision that showed the greatest consequences of not using a normality test, because when model residuals failed the normality test there was a modest improvement in statistical power, 3 to 4%, when switching to a Mann-Whitney test.

### 4.2. Other study considerations

We could have selected other distributions to simulate, but interval data and skewed data are relatively common, and they provided contrast in outcomes. Less common distributions would likely be less applicable to analyses, in addition to many scientists knowing that uncommon and unique distributions require specific error distributions. We wanted our data to be decidedly non-normal, but still common and commonly interpreted as normal.

Statistical transformations, such as logarithmic, arcsine and power transformations, have long been used to address issues of non-normality and heteroscedasticity in data analysis. While these transformations can be useful for predictor variables, their application to response variables has become less encouraged in recent years, in part because it can be hard to understand the mechanistic linkage between a predictor and a transformed variable (for example, the log transform implies that variability in the response is multiplicative on an arithmetic scale rather than additive, which may not be biologically realistic). Instead, statisticians now recommend identifying and modelling the correct underlying distribution that matches the response data. This approach, often implemented through generalized linear models, allows for more accurate representation of the data’s natural structure and avoids potential interpretation difficulties associated with back-transformation. Box and Cox [31] introduced a family of power transformations, but modern statistical methods have evolved to directly model non-normal response distributions, as discussed by Warton and Hui [25]. McCullagh and Nelder [32] provided a comprehensive framework for generalized linear models, which has become the preferred method for handling non-normal response data in many fields.

Lehmann [29] noted that non-parametric tests tend to have about 95% of the power that parametric tests have. In our study, we saw very marginal differences in power. Averaged across all scenarios using interval data, the *t*‐test only detected significant results 1.3% more often than the Mann-Whitney U test (scenarios ranged from 0.3 to 3.7%). Results were flipped for the skewed data simulations—the Mann-Whitney test was about 1% more powerful at detecting significant results (scenarios ranged from 0.3 to −3.0%). Taken together, it is hard to make a case for a clear advantage in statistical power of parametric versus non-parametric tests.

For simplicity, we focused our simulations on two-sample tests of differences in means. However, all linear models (linear regression, analysis of variance, etc.) ultimately depend on the same sum-of-squares calculation of the deviation between the model expectation and residuals. Therefore, it is reasonable to expect that our results would apply generally across the family of linear models, and that we can expect those tests to be robust to deviations from normality, particularly with large effect sizes and sample sizes.

## 5. Conclusion

Normality tests can be extremely useful and powerful statistical tools that help us understand if a sample of data exhibits the statistical properties of normality and thus can be thought of as coming from a normal distribution. Despite the clear purpose of normality tests, misunderstood assumptions of normality in linear regression have led to (possibly widespread) inappropriate applications of normality tests on raw data rather than on model residuals. Although this study strongly recommends the appropriate use of normality tests in linear modelling—which is to evaluate the residuals and not the raw data for normality—our simulations also show that if a normality test is applied to raw data, the subsequent choice of a parametric or non-parametric test has little difference in power. In other words, we found little change in outcomes between a *t*‐test and a Mann-Whitney U test when the raw data were tested for normality. We did observe, however, larger differences in statistical power when normality tests are appropriately used—particularly when a Mann-Whitney test is used after the detection of non-normal residuals, statistical power increases by 3 to 4%. In summary, we can only recommend the appropriate use of a normality test, which remains testing the model residuals for normality and making a decision about a non-parametric test, or another model, based on the residuals. While we strongly recommend proper normality testing of residuals, our findings suggest that incorrect normality testing of raw data—though methodologically flawed—may have little impact on power. This finding does not mean that such practices should be encouraged, but it does suggest that statistical training should shift its focus from overly rigid normality testing to broader principles of model diagnostics and assumption checking. By understanding when statistical mistakes do and do not matter, researchers can prioritize best practices that truly improve inference.

## Data Availability

All data and computer code used to generate the results presented in this study are available online [33]. Supplementary material is available online [34].
